# Multiparametric MRI as an outcome predictor for anal canal cancer managed with chemoradiotherapy

**DOI:** 10.1186/s12885-015-1244-7

**Published:** 2015-04-14

**Authors:** Michael Jones, George Hruby, Peter Stanwell, Sarah Gallagher, Karen Wong, Jameen Arm, Jarad Martin

**Affiliations:** 1 Radiation Oncology, Royal Prince Alfred Hospital, Salisbury Road, Camperdown, NSW 2050 Australia; 2 Radiation Oncology, Chris O’brien Lifehouse, Missenden Road, Camperdown, NSW 2050 Australia; 3 Faculty of Health and Medicine, University of Newcastle, Callaghan, NSW 2308 Australia; 4 Calvary Mater Newcastle, Edith Street, Waratah, NSW 2298 Australia; 5 Radiation Oncology, Liverpool Hospital, Corner of Elizabeth and Goulburn Streets, Liverpool, NSW 2170 Australia; 6 Radiation Oncology, Calvary Mater Newcastle, Edith Street, Waratah, NSW 2298 Australia

**Keywords:** Anal neoplasms, Squamous Cell Carcinoma or SCC, Magnetic Resonance Imaging or MRI, Chemoradiotherapy, Diffusion weighted imaging or DWI, Dynamic contrast enhanced or DCE

## Abstract

**Background:**

Organ-preserving chemo-radiotherapy (CRT) is the standard of care for non-metastatic anal squamous cell carcinoma (SCC). The optimal dosing schedules are yet to be determined. To improve local control rates, dose escalation has been investigated but found to not increase efficacy at the expense of increased toxicity for an unselected patient population.

Diffusion weighted imaging (DWI) and dynamic contrast enhanced (DCE) Magnetic Resonance Imaging (MRI) performed during CRT have early data suggesting it to be an effective tool in predicting later tumour response for SCC in related body sites.

By performing multi-parametric MRI (mpmMRI) incorporating standard morphological, DWI and DCE sequences, we aim to determine whether the early changes in multi-parametric parameters during CRT can predict for later response in anal SCC. This may create opportunities to investigate treatment adaptation, either intensification or de-escalation, during CRT.

**Methods/Design:**

This protocol describes a prospective non-interventional multi-centre single-arm clinical trial. Twenty eligible patients with histologically confirmed non-metastatic anal SCC will receive standard definitive CRT and undergo multi-parametric MRI’s at the following 4 time points; prior to treatment, during the second and fourth weeks of treatment and 6-8 weeks following treatment.

Complete response will be defined by the absence of tumour persistence or recurrence as determined by clinical examination at 6 months.

Images will be retrospectively analysed to determine the apparent diffusion coefficient and tumour perfusion coefficients (K_trans_ and K_ep_) at each time point. The Mann-Whitney-Wilcoxon Test will be utilised to compare the change in these parameters for responder’s verses non-responders.

**Discussion:**

If validated, mpmMRI, along with other risk factors, can be used to stratify patients and guide radiation dosing in a prospective trial. Informed individualisation of treatment intensity should help us achieve our goals of improved efficacy and reduced toxicity.

**Trial registration:**

Australian New Zealand Clinical Trials Registry (ANZCTR): ACTRN12614001219673 (19/11/2014).

## Background

### Anal cancer

#### Prevalence and risk factors

Anal cancer (AC) is an uncommon malignancy, representing 2.2% of all gastrointestinal cancers. However, the rate of AC is increasing [1]. This is likely due to the rising prevalence of its strongest risk factors – Human Papilloma Virus (HPV) and Human Immunodeficiency Virus (HIV) [2-5]. The majority of anal squamous cell carcinomas (SCCs) are associated with HPV infection and, in particular, the HPV-16 subtype [3,6].

#### Treatment

Pioneering data from the 1970s found that combined chemo-radiotherapy (CRT) could achieve a complete response in anal cancer [7]. Radical CRT has since become the standard of care for non-metastatic AC [8]. The long-standing combination of radiotherapy, 5-Fluorouracil (5-FU) and Mitomycin-C (MMC) has been validated in a series of large prospective randomised controlled trials [9-13].

### Multiparametric MRI

#### Diffusion Weighted MRI (DW-MRI)

DW-MRI is a functional MRI technique that measures molecular diffusion resulting from normal Brownian motion of water protons within biological tissues [14]. Due to architectural differences, biological tissues are variably restrictive of diffusion. In particular, the densely cellular and disorganised architecture characteristic of cancer results in low molecular diffusion and therefore low signal response. Diffusion is measured quantitatively by the apparent diffusion coefficient (ADC).

#### Dynamic Contrast Enhanced MRI (dCE-MRI)

DCE-MRI is performed by obtaining sequential MRI images acquired before, during and after intravenous injection of paramagnetic contrast [15]. DCE-MRI measures the rate of contrast movement between the intravascular and extra-cellular extravascular space. This rate reflects tissue microvasculature permeability and perfusion. Cancer, with its abnormal neovasculature, tends to show characteristic changes in the signal intensity compared with normal tissues [16].

### Rationale for the proposed study

#### Multiparametric MRI as a biomarker in anal cancer

Presently, CRT results in local failure rates of 14 to 37%, even in patients with early stage disease [17-19]. Although it is feasible to intensify the radiotherapy dose, this increases toxicity, and a recent randomized trial has shown that this dose escalation strategy is not beneficial for an unselected patient population [10].

Nigro et al. reported local control in 23 of 28 patients who received an intermediate radiation dose of 30 Gy in 15 fractions combined with 1000 mg/m^2^ of 5-FU delivered on days 1-5 and 29-33, and 15 mg/m^2^ of Mitomycin-C on day 1 only [20]. More recently, Hu et al. [21] and Hatfield et al. [22] found 30Gy sufficient to treat microscopic and small volume (<2 cm) macroscopic disease.

This suggests there are some anal cancers that require less than the current regimen of approximately 50-55 Gy, and others that require more. Adaptive radiotherapy using a biomarker would allow clinicians to tailor the treatment dose to an individual patient’s tumour response. Such a strategy could see decreased toxicity (perineal skin atrophy and fibrosis, sexual dysfunction, femoral neck fractures and persistent gastrointestinal disturbance such as sphincter dysfunction) and improved local control rates.

Diffusion-weighted MRI (DW-MRI) and dynamic contrast enhanced MRI (dCE-MRI) performed during CRT have early data suggesting it to be an effective tool in predicting later tumour response for both cervical and head & neck cancer [23,24]. As cervical and some head and neck SCCs share a common aetiology with anal SCC via Human Papilloma Virus (HPV), there are grounds to hypothesize that similar tumour response patterns may occur across these various malignancies when treated with organ preserving CRT. Application of this modality to anal cancer may allow adaptation of radiotherapy dosing, both intensification and de-escalation, to compensate for observed tumour biology.

### Study hypothesis

DW-MRI and dCE-MRI performed during CRT for AC is predictive of later tumour response and prognostic of outcome.

## Methods

### Study design

This study is designed to be a single-arm, multicenter, prospective, observational trial to investigate the value of mpmMRI as an imaging biomarker in AC for later response to CRT.

### Objectives

#### Primary study endpoint

Correlation of mpmMRI parameters with tumour response.

#### Secondary study endpoint

Determine the feasibility of performing mpmMRI during CRT for AC.

#### Study schematic

**Figure 1:**
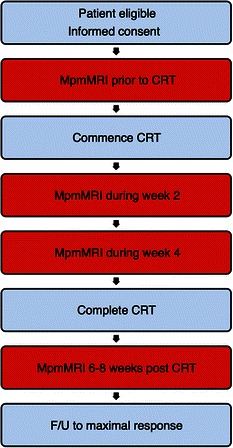
Study Schematic. MpmMRI = Multi-parametric magnetic resonance imaging, CRT = Chemo-radiotherapy, F/U = Follow-up.

All patients will receive standardized CRT and have mpmMRI performed at the four time points of pre-treatment, weeks 2 and 4 of CRT, and then 6-8 weeks following completion of CRT (Figure 1).

### Subject selection and withdrawal

#### Inclusion criteria

1. Patient capable of providing informed consent
2. Patient deemed suitable for protocol treatment as assessed by Radiation and Medical Oncologists
3. Histological diagnosis of invasive primary squamous cell carcinoma of the AC
4. TNM Stage: T2-4 N0-3 M0 based on the following diagnostic work-up
  - History & physical examination including:
    i. Digital Rectal Exam (DRE) stating primary size and distance from anal verge
    ii. Groin examination with documentation of any lymphadenopathy
  - Anal biopsy
  - CT abdomen and pelvis
  - Whole Body PET/CT
5. Age ≥18

#### Exclusion criteria

1. ECOG performance status >2
2. Significant comorbidities that would interfere with the completion of treatment
3. Renal insufficiency (Creatinine > 150)
4. Prior radiotherapy to the pelvis
5. Prior surgery for cancer of the anus that removed all macroscopic cancer
6. Prior systemic chemotherapy for anal cancer
7. Evidence of distant metastases (M1) if this precludes radical pelvic treatment
8. Women who are pregnant or lactating
9. Inability to have a MRI due to:
  - Implanted magnetic metal e.g. intraocular metal
  - Pacemaker/Implantable defibrillator
  - Extreme claustrophobia

### Radiation therapy

The radiation technique must be one of either:

- Intensity Modulated Radiation Therapy (IMRT)
- Volumetric Modulated Arc Therapy (VMAT)
- Tomotherapy

The treatment plan is at the discretion of the treating Radiation Oncologist and should be determined by analysis of the volumetric dose, the Dose Volume Histograms, planning target volume (PTV) and critical normal structures. An “inverse” planning method will be used with the aim of delivering dose to the PTV while maximally sparing the normal tissues.

#### Target prescription dose

Dose and fractionation for radical treatment is guided by the Australasian Gastrointestinal Trials Group (AGITG) guidelines [25]. The target prescription dose shall be determined as follows:

##### For T2N0 disease

- The primary tumour PTV will receive 50.4 Gy in 28 fractions at 1.8 Gy per fraction
- The uninvolved nodal PTVs will receive 42 Gy in 28 fractions at 1.5 Gy per fraction

##### For T3-4 N0 disease

- The primary tumour PTV will receive 54 Gy in 30 fractions at 1.8 Gy per fraction.
- The uninvolved nodal PTVs will receive 45 Gy in 30 fractions at 1.5 Gy per fraction.

##### For N1-3 disease:

- The primary tumour PTV will receive 54 Gy in 30 fractions at 1.8 Gy per fraction.
- For involved nodes ≤ 3 cm in maximum dimension, the involved nodal PTV will receive 50.4 Gy in 30 fractions at 1.68 Gy per fraction.
- For involved nodes > 3 cm in maximum dimension, the involved nodal PTV will receive 54 Gy in 30 fractions at 1.80 Gy per fraction.

#### Dose specifications

The following dose specifications are recommended:

- 98% of the relevant PTV should receive >95% of the prescription dose
- No more than 2% of the relevant PTV should receive >107% of the prescription dose

#### Treatment schedule

Treatment will be delivered once daily on weekdays, 5 days per week except on public holidays. Missed fractions will be made up for at the end of treatment at the discretion of the treating clinician. All PTVs will be treated simultaneously. Treatment breaks will be avoided, if possible, or minimised.

#### Treatment planning

Target volume definitions are as per ICRU Reports 50, 62 and 83. Treatment planning is as per the Australasian Gastrointestinal Trials Group (AGITG) Contouring Atlas and Planning Guidelines for Intensity-Modulated Radiotherapy in Anal Cancer [25]. This will include elective nodal irradiation of the mesorectum, presacral space, ischiorectal fossa, inguinal, obturator, internal and external iliac lymph nodes,

##### Gross tumour volumes (GTV)

The gross disease is determined by physical examination, CT, PET and/or MRI.

##### Clinical target volume (CTV)

The primary CTV must encompass:

1. GTV
2. Entire anal canal from the ano-rectal junction to the anal verge
3. Internal and external anal sphincters

A further 10-20 mm isotropic margin should be added to items (1), (2), and (3) above, to account for microscopic disease, while respecting anatomical boundaries. For the involved nodes or nodal regions, a 10-20 mm margin should be used, respecting anatomical boundaries.

##### Planning target volume (PTV)

An isotropic 10 mm expansion is recommended on CTVs to generate PTVs. Daily image guidance is recommended, which may allow CTV-PTV margin reduction to 5-7 mm.

#### Dose constraints

The following normal tissue dose constraints are recommended. Where available, values are taken from the QUANTEC papers. Where not available for that organ, dose constraints are listed as per the RTOG 0529 closed study protocol (Table 1).

**Table 1:** **Recommended organ at risk dose constraints**

ORGAN	CONSTRAINTS: No More than		
**Small Bowel**	195 cc above 45 Gy	1% of small bowel > 52 Gy	
**Femoral Head**	50% above 30 Gy	35% above 40 Gy	5% above 44 Gy
**Iliac Crests**	50% above 30 Gy	35% above 40 Gy	5% above 50 Gy
**External Genitalia**	50% above 20 Gy	35% above 30 Gy	5% above 40 Gy
**Bladder**	50% above 55 Gy		
**Large Bowel**	50% above 50 Gy		

### Chemotherapy

Concurrent chemotherapy will begin on the first day of radiotherapy. The second course of chemotherapy will commence on calendar day 29 [4,9].

#### 5-Fluorouracil (5-FU)

5-FU shall be delivered at a dose of 800-1000 mg/m^2^/day via the IV route for 96 hours continuously starting on day 1 and repeated on day 29. In the instance of an unplanned treatment break, the second cycle of 5-FU shall be delivered on the 29^th^ day of radiotherapy treatment.

#### Mitomycin-C

Mitomycin-C shall be delivered at a dose of 10 mg/m^2^ (without exceeding a maximal single dose of 20 mg) via the IV route on day 1 +/- day 29, depending on local practice.

### Pathology

All biopsy tissues will be formalin fixed, paraffin embedded and routine H&E stained. Immunohistochemical p16 staining is to be performed for all tumours as recent data suggests that p16 positivity correlates with HPV status and is associated with reduced relapse rates and improved overall survival [26].

### Follow-up and surgery

At 6-8 weeks post CRT, the patient will have a mpmMRI performed.

The follow-up schedule is at the discretion of the treating clinician. However, the following suggestions apply:

#### Progressive disease

- Biopsy

- ○ If negative, reassess in 4 weeks
- ○ If positive and no evidence of distant disease, consideration of abdominoperineal resection (APR) is recommended

#### Persistent disease

- No biopsy, reassess in 4 weeks
- Patients with clinical suspicion of persistent disease at 26 weeks should undergo a biopsy and consideration of APR, if positive.

#### Complete clinical response

- No biopsy
- Continue to follow-up at the discretion of treating clinician

### Imaging

#### Imaging schedule

MpmMRI consists of standard morphological MRI, DW-MRI and dCE-MRI. Patients will undergo mpmMRI at the following four time points:

- Prior to CRT
- During the second week of treatment (fraction days 6-10)
- During the fourth week of treatment (fraction days 16-20)
- At 6-8 weeks post treatment

#### Imaging process

MRI’s are performed on a 3 Tesla device. Patients are scanned in the supine position. No rectal coil is used. All patients should have a single IV bolus of Buscopan (20 mg/ml) immediately prior to the first sequence.

##### Diffusion weighted imaging

- Performed at 4 b-values
  - ○ 0, 400, 800 and 1200

##### Dynamic contrast enhanced imaging

- Contrast injection:
  - ○ Magnevist 0.2 ml/kg
  - ○ Power Injector (2.5 ml/s)
  - ○ 20 ml saline chase at same rate as injection

The eGFR must be checked prior to the MRI to ensure eligibility for full contrast injection. Half doses are not permitted.

#### Imaging analysis

##### Standard morphological MRI (SM-MRI)

All images will be assessed independently by two radiologists to determine primary and nodal tumour dimensions. Where there is disagreement, a third will be asked to mediate.

##### Diffusion weighted MRI (DW-MRI)

A Region Of Interest (ROI) will be placed over primary and involved nodal regions to calculate mean and median primary and nodal ADC values

##### Dynamic contrast enhanced MRI (dCE-MRI)

**Figure 2:**
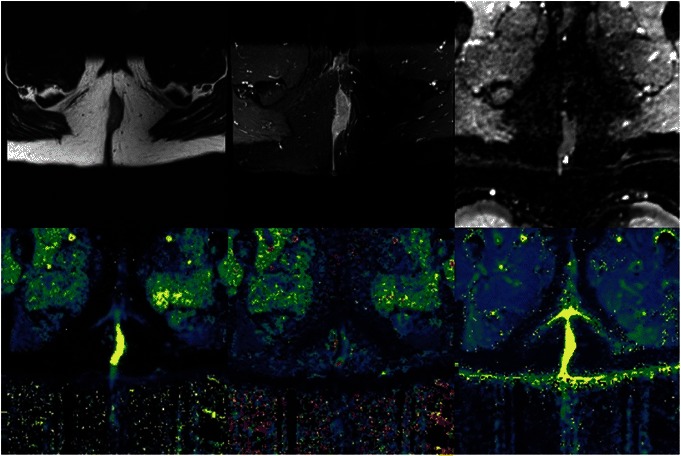
Anal cancer. Morphological MRI (top left and top middle), apparent diffusion coefficient map (top right), dynamic contrast enhanced parametric maps (bottom, from left to right): K_Trans_, K_ep_ and V_e_.

- A ROI will be placed over the entire primary and involved nodal regions to calculate mean and median primary and nodal K^trans^ and K_ep_ values and Relative Signal Intensity (RSI) (Figure 2)

### Statistical considerations

#### Sample size determination

Assuming that 70% of patients are positive responders, then sample sizes of 14 responders and 6 non-responders will achieve between 70% and 80% power to show a difference in mean change (initial to final) in SM-MRI of between 1.2 and 1.4 standard deviations at the 0.05 significance level (alpha) using a two-sided Mann-Whitney-Wilcoxon Test. Previous studies in other body locations have shown a positive result with similar patient numbers. We anticipate recruitment to be achieved within 24 months.

#### Definition of complete response

- No evidence of residual tumour at 26 weeks post CRT
- No progression requiring APR prior to 26 weeks

### Ethical considerations

This protocol along with the informed consent document and patient information sheet has received ethics approval from the Hunter New England Human Research Ethics Committee (HREC). The protocol also has radiation safety approval.

### Study finances

This study has been funded by both the Hunter Translational Cancer Research Unit (HTCRU) and the Royal Australian and New Zealand College of Radiologists (RANZCR), each with a $20,000 competitive research grant (Total = $40,000). Neither the HTCRU nor the RANZCR have been involved in the writing of this protocol or will have any influence on the analysis or publication of the study.

## Discussion

There is no consensus on the optimal radiation dose for the treatment of patients with AC. It is very likely that small tumours are often over-treated and large tumours sometimes under-treated. Although TNM staging is highly prognostic for AC, there is still significant heterogeneity in outcomes within a particular stage. Improved prognostication may be achieved with further information such as HPV status and mpmMRI tumour response. If this exploratory phase 2 study finds compelling evidence for an imaging biomarker being independently predictive of later tumour response, a subsequent study would aim to validate this by intensifying radiotherapy dose for tumours with unfavourable biology, and deescalating radiotherapy dose for favorable tumours. If validated, an imaging biomarker for response to CRT would allow clinicians to adapt and personalise treatment, which holds the potential for improved efficacy and reduced toxicity.

## Abbreviations

5-FU: 5-fluorouracil
AC: Anal cancer
ADC: Apparent diffusion coefficient
AGITG: Australasian gastro-intestinal trials group
APR: Abdomino-pelvic resection
CRT: Chemo-radiotherapy
CT: Computed tomography
CTV: Clinical target volume
DCE: Dynamic contrast enhanced
DRE: Digital Rectal examination
DWI: Diffusion weighted imaging
ECOG: Eastern cooperative oncology group performance status
eGFR: Estimated glomerular filtration rate
GTV: Gross tumour volume
HIV: Human influenza virus
HPV: Human papilloma virus
HREC: Human resource ethics committee
HTCRU: Hunter translational cancer research unit
IMRT: Intensity modulated radiotherapy
MMC: Mitomycin-C
mpmMRI: Multi-parametric MRI
MRI: Magnetic resonance imaging
NS: Normal saline
PET: Positron emission tomography
PICC: Peripherally inserted central catheter
PTV: Planning target volume
QUANTEC: Quantitative analyses of normal tissue effects in the clinic
RANZCR: Royal Australian and New Zealand college of radiologists
ROI: Region of interest
RSI: Relative signal intensity
RTOG: Radiation therapy oncology group
SCC: Squamous cell carcinoma
SIA: Subject identification number
SM: Standard morphological
VMAT: Volumetric modulated arc therapy
